# Emphysematous Cystitis in an Immunocompromised Patient With Ovarian Cancer: A Case Report

**DOI:** 10.7759/cureus.92863

**Published:** 2025-09-21

**Authors:** Kole Winebrenner, Madeline Manuel, John Greene

**Affiliations:** 1 Department of Infectious Diseases, Nova Southeastern University Dr. Kiran C. Patel College of Osteopathic Medicine, Clearwater, USA; 2 Department of Infectious Diseases, Nova Southeastern University Dr. Kiran C. Patel College of Osteopathic Medicine, Fort Lauderdale, USA; 3 Department of Internal Medicine, Division of Infectious Diseases, Moffitt Cancer Center, Tampa, USA

**Keywords:** carboplatin, complicated urinary tract infection, conservative management, corticosteroid-induced immunosuppression, ct imaging diagnosis, emphysematous cystitis, klebsiella pneumoniae, opioid-induced urinary retention, ovarian cancer, pacitaxel

## Abstract

Emphysematous cystitis (EC) is a rare, potentially life-threatening infection characterized by gas formation within the bladder wall or lumen, most commonly caused by *Escherichia coli* or *Klebsiella pneumoniae*. It is typically associated with diabetes mellitus, immunosuppression, or urinary tract abnormalities. We present a case of EC in a 67-year-old immunocompromised female with advanced-stage ovarian cancer, undergoing chemotherapy with carboplatin and paclitaxel at the time of diagnosis. Her additional risk factors included diabetes mellitus and chronic corticosteroid use. Diagnosis was established via CT angiography, which revealed extensive gas within the bladder wall. Urine culture grew *Klebsiella pneumoniae* sensitive to ceftriaxone, which was administered intravenously. The patient was managed conservatively with antibiotics, bladder decompression, and supportive care, resulting in resolution of the infection. To our knowledge, this is the first reported case of EC in a patient receiving carboplatin and paclitaxel chemotherapy for ovarian cancer, underscoring the diagnostic complexity and management considerations of EC in oncology patients with compounding immunosuppressive risk factors.

## Introduction

Emphysematous cystitis (EC) is a rare, potentially life-threatening form of complicated urinary tract infection (UTI) characterized by gas accumulation within the bladder wall or lumen due to gas-forming organisms, most commonly *Escherichia coli* and *Klebsiella pneumoniae* [1]. It primarily affects women over 60 years old, with diabetes mellitus being the most frequently associated risk factor [1]. Other predisposing conditions include bladder dysfunction, malignancy with chemotherapy, chronic corticosteroid use, and recent urologic surgery [1]. Clinical presentations of EC may vary significantly. In a literature review of 113 cases, sepsis was the most common presenting symptom (33.7%), followed by abdominal pain (25.7%), and typical UTI symptoms (16.8%) [1]. Diagnosis is primarily radiographic, with CT with or without contrast being the most sensitive study for detecting intramural or intraluminal gas [1]. Early diagnosis, empiric antibiotics followed by culture-guided adjustments, and bladder decompression is essential to prevent progression and achieve favorable outcomes. Oncology patients, particularly those receiving chemotherapy or chronic corticosteroid therapy, represent a distinct diagnostic challenge because their immunosuppressed state and atypical presentations can delay recognition of EC.

## Case presentation

A 67-year-old female with high-grade serous ovarian carcinoma (PAX8+, WT1+, p53+, BRCA1/2+) and peritoneal metastases, complicated by steroid-induced hyperglycemia and coronary artery disease with multiple stent placements, was admitted for evaluation of acute upper gastrointestinal bleeding with melanotic stools, chronic oropharyngeal candidiasis with suspected esophagitis, and acute-on-chronic dysuria. She was receiving chemotherapy with carboplatin and paclitaxel at the time and was later transitioned to maintenance olaparib. The patient reported intermittent dysuria, hematuria, and urinary straining for several months. Although pneumaturia is a classic symptom of EC [1], it was not observed in this case. In the weeks preceding admission, her daily prednisone dose had increased from 10-15 mg to 20 mg for management of cancer-related pain and inflammation. This regimen was clinically indicated but contributed to her steroid-induced hyperglycemia and further immunosuppression.

On initial evaluation, she was afebrile, hemodynamically stable, and pancytopenic. Laboratory tests revealed a blood glucose level of 322 mg/dL and a hemoglobin A1c of 8.1%, consistent with poorly controlled steroid-induced hyperglycemia. Additionally, her lactate level was elevated at 2.4 mmol/L. Blood cultures were not documented during this admission because the patient remained hemodynamically stable, and clinical suspicion for bacteremia was low. Follow-up hematology and biochemistry values remained stable and are therefore not detailed here, as the admission results were sufficient to guide diagnosis and management. Subsequent urinalysis was notable for significant glucosuria (≥1,000 mg/dL), hematuria, proteinuria, positive nitrites, 1+ bacteria, and the presence of yeast. Empiric intravenous ceftriaxone was initiated before the Infectious Disease consultation. In the weeks preceding admission, the patient experienced recurrent urinary symptoms. A urine culture during this period grew *Klebsiella pneumoniae*, for which she received a short, incomplete course of amoxicillin-clavulanate that provided only transient relief, and her symptoms recurred in a similar pattern, ultimately progressing to EC. She also had a history of inconsistent use of opioids, including oxycodone, hydrocodone-acetaminophen, and tramadol, which may have contributed to her urinary retention. A postvoid residual bladder scan revealed 650 mL of retained urine, consistent with acute urinary retention, which was managed with Foley catheter placement. This intervention alleviated her suprapubic pain, and the catheter was planned to be removed during inpatient rehabilitation; however, the exact timing of removal was not documented in the inpatient record.

**Figure 1:**
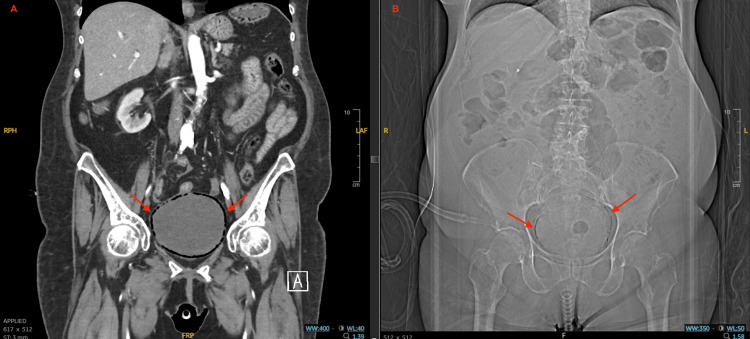
Abdominal coronal CT angiography and abdominal X-ray – emphysematous cystitis. (A) Coronal CT angiography of the abdomen and pelvis showing intramural bladder wall gas (red arrows) consistent with emphysematous cystitis. (B) Abdominal X-ray demonstrating mottled radiolucencies overlying the bladder (red arrows), corresponding to gas within the bladder wall. While X-ray can detect intramural air, its sensitivity and anatomical detail are inferior to CT.

**Figure 2:**
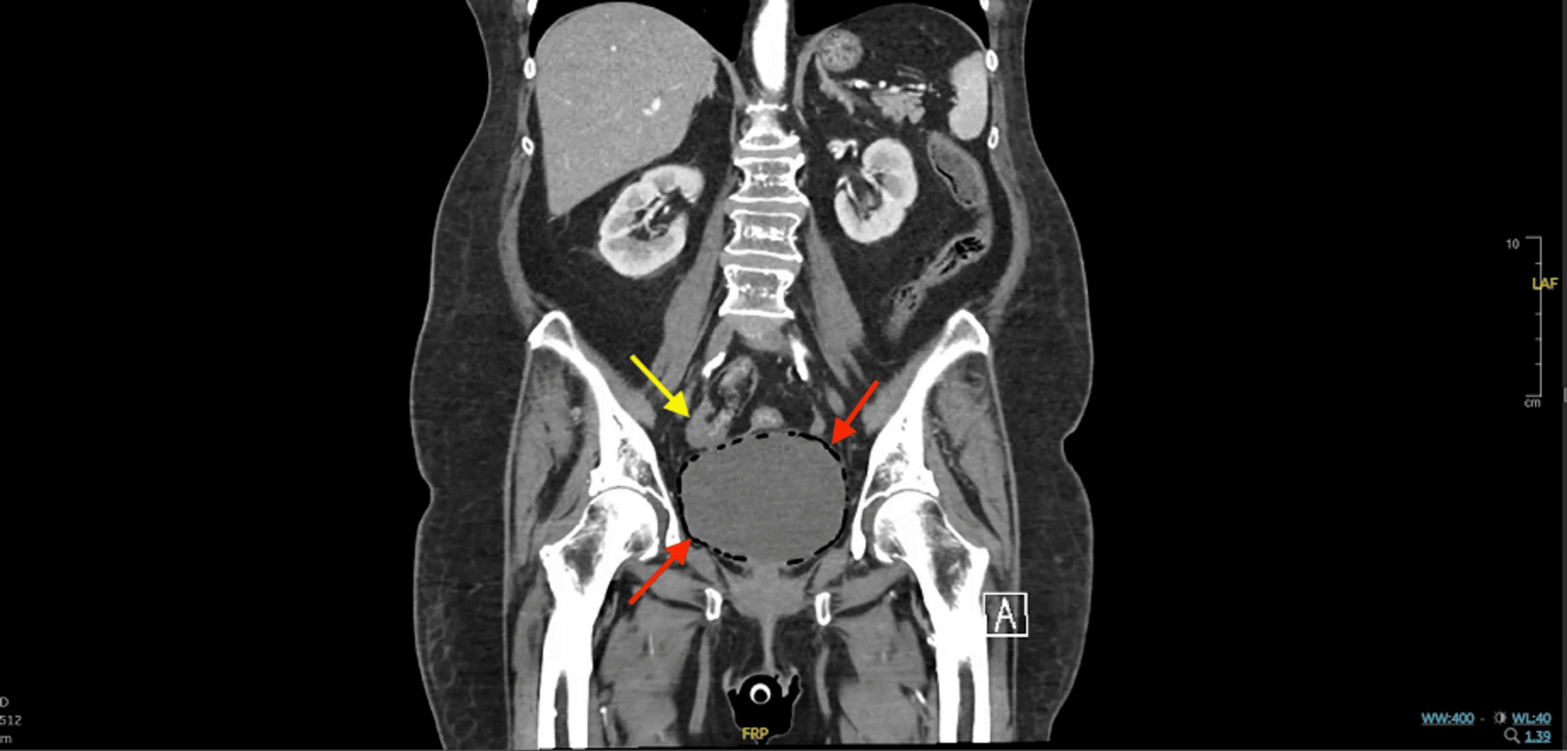
Coronal CT angiography – emphysematous bladder wall gas. Coronal CT angiography of the abdomen and pelvis demonstrating diffuse gas within the bladder wall (red arrows) without extension into the renal collecting system, thereby ruling out emphysematous pyelonephritis. The right ovary (yellow arrow) lies adjacent to the bladder wall with no evidence of fistula, abscess, or necrosis.

**Figure 3:**
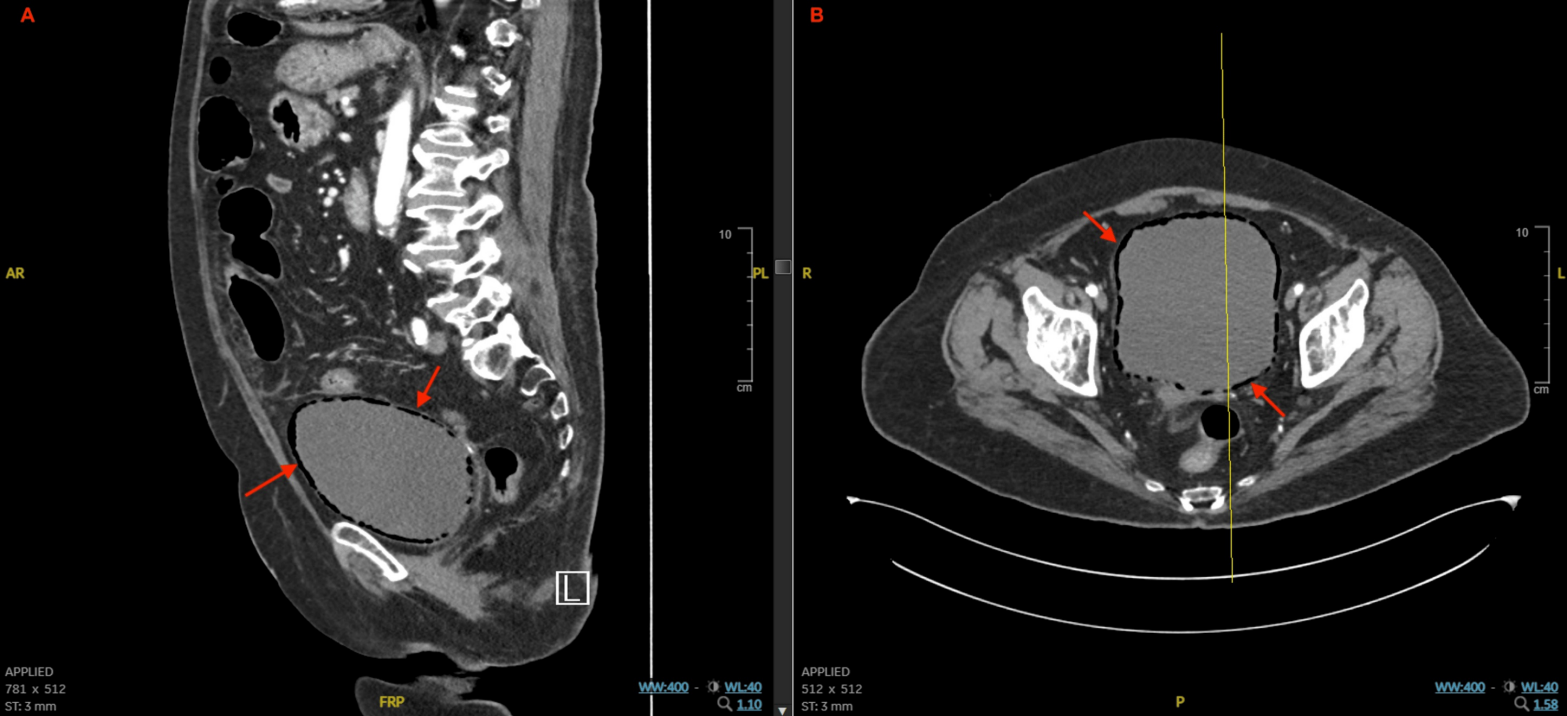
Sagittal and axial CT views of emphysematous cystitis. (A) Sagittal CT angiography showing gas tracking circumferentially within the bladder wall. (B) Axial CT angiography confirming intramural gas throughout the bladder wall. No extraluminal extension or associated abscess is observed.

Although her urinary symptoms were initially presumed to be due to a recurrent uncomplicated UTI, a CT angiography of the abdomen and pelvis obtained to evaluate for gastrointestinal hemorrhage incidentally revealed extensive intramural gas within the bladder wall. Figure 1 demonstrates intramural air on both X-ray and coronal CT, while Figure 2 and Figure 3 depict coronal, sagittal, and axial views illustrating the extent and circumferential distribution of gas without upper tract involvement, findings characteristic of EC. Based on culture sensitivities, intravenous ceftriaxone was continued for 14 days. She was discharged to a rehabilitation facility to complete the antibiotic course, with plans to transition to oral trimethoprim-sulfamethoxazole for an additional seven days if catheterization remained necessary. Subsequent hospitalizations for unrelated reasons, during which imaging was performed, showed no evidence of EC recurrence. A summary of the patient’s comorbidities, key laboratory findings, and management is provided in Table 1.

**Table 1:** Summary of patient’s comorbidities, key laboratory findings, and management. TMP-SMX = trimethoprim-sulfamethoxazole

Category	Findings
Comorbidities	High-grade serous ovarian carcinoma with peritoneal metastases; coronary artery disease with stent placements; steroid-induced hyperglycemia; chronic corticosteroid use; chronic oropharyngeal candidiasis; opioid use with urinary retention
Laboratory values	Blood glucose (322 mg/dL); hemoglobin A1c (8.1%); lactate (2.4 mmol/L); urinalysis: glucosuria (≥1,000 mg/dL); moderate hematuria; proteinuria; nitrites positive; 1+ bacteria; yeast present
Management	IV ceftriaxone (14 days); Foley catheter bladder decompression; planned oral TMP-SMX step-down; supportive care

## Discussion

Uncomplicated UTIs affect more than 150 million people worldwide each year and account for over 10 million annual outpatient visits in the United States, making them one of the most common bacterial infections [2]. In contrast, complicated UTIs, such as EC, are far less prevalent but carry a mortality rate of about 7% in hospitalized patients [2]. This case highlights the clinical variability of EC and the importance of early recognition, especially in immunocompromised hosts. However, diagnostic pitfalls are common, as presenting urinary symptoms are often nonspecific, classic signs such as pneumaturia may be absent, and overlapping oncologic comorbidities can delay recognition [1,3].

While diabetes mellitus is the most well-established risk factor reported, other contributors include chronic urinary retention, recurrent urinary infections, indwelling catheters, and immunosuppressive states [1]. In this case, our patient had multiple overlapping risk factors, including ovarian cancer, recent cytotoxic chemotherapy, corticosteroid use, and bladder dysfunction.

The exact pathophysiology of EC is incompletely understood. While gas-forming organisms such as *Escherichia coli* and *Klebsiella pneumoniae* are the most common pathogens, their gas production alone does not fully explain the disease. Proposed mechanisms include bacterial fermentation of urinary substrates such as glucose, proteins, and lactulose, particularly in patients with glucosuria or hyperglycemia [3,4]. On admission, our patient had a blood glucose of 322 mg/dL, hemoglobin A1c of 8.1%, and significant glucosuria (≥1,000 mg/dL), indicating poorly controlled hyperglycemia. Additional findings included a lactate of 2.4 mmol/L and significant glucosuria (≥1,000 mg/dL), which support this proposed mechanism. Other urinalysis findings included proteinuria, hematuria, bacteruria, and nitrites, reflecting significant urinary tract involvement.

Additionally, opioid-induced urinary stasis may have also contributed to infection risk [5]. Although her use of oxycodone, hydrocodone-acetaminophen, and tramadol was inconsistent, opioids are known to impair detrusor contractility and increase urethral sphincter tone, promoting urinary retention and providing a favorable environment for bacterial proliferation [5]. Another theory suggests that intramural gas accumulation may cause pressure-induced necrosis of local tissue, which creates a favorable protein-rich environment for further bacterial growth and impairs gas diffusion [3]. A third proposed mechanism involves inflammatory infiltrate and bacterial toxins increasing bladder wall permeability, facilitating gas invasion into the tissue [6].

This case illustrates the importance of maintaining a high index of suspicion for EC in non-diabetic immunocompromised patients. Our patient’s ovarian cancer, chemotherapy with carboplatin and paclitaxel, chronic corticosteroid therapy, and urinary retention requiring catheterization appear to have compounded her infection risk. Patients with advanced ovarian cancer may be predisposed to UTIs due to pelvic tumor burden, bladder dysfunction, and systemic immunosuppression [1,7,8]. In addition, carboplatin and paclitaxel are well known to cause myelosuppression, including neutropenia and pancytopenia, which significantly increase susceptibility to opportunistic infections [9,10]. Early CT imaging and urine culture-directed antibiotic therapy were essential in achieving a favorable outcome. Oncology patients undergoing active treatment may present atypically, and early intervention is critical to avoid progression to emphysematous pyelonephritis (EP) or sepsis. In this case, the patient remained afebrile and hemodynamically stable despite advanced infection, an atypical feature often seen in oncology patients with immunosuppression, which may delay recognition and diagnosis.

Role of imaging in immunocompromised hosts

In patients with diminished immunity, classic signs of infection, such as fever, leukocytosis, and urinary tract symptoms, may be less apparent or completely absent [3], delaying the diagnosis of EC. While ultrasound and X-ray can identify intramural air, CT remains the gold standard, offering superior sensitivity and anatomical definition [1], enabling timely diagnosis, evaluation of disease extent, and exclusion of complications such as fistula, abscess, or bladder wall necrosis. In this case, CT angiography was obtained to evaluate for potential gastrointestinal hemorrhage, but incidentally revealed the characteristic findings of EC. This underscores the diagnostic utility of cross-sectional imaging in immunocompromised patients, even when ordered for unrelated indications.

Conservative management versus surgical intervention

Although EC with bladder necrosis or EP may require cystectomy or nephrectomy [4], most uncomplicated cases can be managed conservatively. This includes specific antibiotics based on culture sensitivities, temporary bladder decompression via straight catheterization or Foley placement, glycemic control, and eventual removal of chronic indwelling catheters to reduce recurrence of infection [1,4].

Our patient’s successful recovery with conservative management aligns with outcomes in immunocompromised patients described in other cases. However, few reports document EC specifically in patients with ovarian cancer. Sereno et al. described a breast cancer patient on chemotherapy, successfully treated with three weeks of piperacillin-tazobactam [7]. Another report by Tetsuya et al. involved a lung cancer patient receiving carboplatin, paclitaxel, and dexamethasone who developed both EC and EP due to *Klebsiella pneumoniae* that responded to meropenem, levofloxacin, and urethral stent placement [8]. Notably, our patient received the same chemotherapeutic agents in combination with chronic steroid use, further highlighting a similar risk profile.

Emerging infections in the era of targeted cancer therapies

Cytotoxic chemotherapy is a well-established risk factor for infection due to its myelosuppressive effects [9]. In this case, the patient was undergoing treatment with carboplatin and paclitaxel at the time of her EC diagnosis, agents known to cause neutropenia, anemia, and lymphopenia [10]. These hematologic toxicities significantly weaken host defenses and predispose patients to opportunistic infections [9,10]. This vulnerability extends to the urinary tract, where immunosuppression and neutropenia may facilitate the development of severe infections such as EC [1,7,8]. This patient’s immunosuppressive state was further compounded by chronic corticosteroid use and mucosal barrier injury from candidal esophagitis.

Although olaparib had not yet been initiated, it was later prescribed due to the patient’s BRCA1/2 mutation. While poly(ADP-ribose) polymerase inhibitors such as olaparib are not traditionally considered immunosuppressive, they are associated with hematologic toxicity, including neutropenia and lymphopenia, which may further increase infection susceptibility [10,11]. Despite these risks, olaparib has been shown to improve progression-free survival in patients with advanced ovarian cancer [11].

This case highlights the importance of recognizing the cumulative immunosuppressive burden of cancer-directed therapies. As targeted agents become more widely prescribed, clinicians must remain aware of atypical infections such as EC, especially in patients with overlapping risk factors.

## Conclusions

To our knowledge, this is the first reported case of EC in a patient receiving carboplatin and paclitaxel chemotherapy for ovarian cancer, where chemotherapy-associated cytopenias, chronic corticosteroid use, and hyperglycemia likely compounded her immunosuppressed state and contributed to infection risk. This case highlights the need to consider EC in immunocompromised oncology patients who present with subtle urinary symptoms, urinalysis findings suggestive of infection, a urine culture positive for gas-forming organisms, or signs of urosepsis. While the observations from a single case cannot be broadly generalized, this report underscores the importance of maintaining clinical suspicion and demonstrates that conservative management with intravenous antibiotics and bladder decompression can be effective in achieving complete resolution, even in complex oncology patients. Timely CT imaging remains essential for diagnosis, along with early antimicrobial therapy and management of contributing comorbidities, including hyperglycemia and chronic corticosteroid use. Ongoing urologic monitoring and risk factor modification are important to prevent recurrence.
